# Comparing treatment strategies to reduce antibiotic resistance in an in vitro epidemiological setting

**DOI:** 10.1073/pnas.2023467118

**Published:** 2021-03-25

**Authors:** Daniel C. Angst, Burcu Tepekule, Lei Sun, Balázs Bogos, Sebastian Bonhoeffer

**Affiliations:** ^a^Institute of Integrative Biology, Department for Environmental System Science, ETH Zurich, 8092 Zurich, Switzerland

**Keywords:** combination therapy, antibiotic resistance, experimental epidemiology

## Abstract

The rapid rise of antibiotic resistance, combined with the increasing cost and difficulties to develop new antibiotics, calls for treatment strategies that enable more sustainable antibiotic use. The development of such strategies, however, is impeded by the lack of suitable experimental approaches that allow testing their effects under realistic epidemiological conditions. Here, we present an approach to compare the effect of alternative multidrug treatment strategies in vitro using a robotic liquid-handling platform. We use this framework to study resistance evolution and spread implementing epidemiological population dynamics for treatment, transmission, and patient admission and discharge, as may be observed in hospitals. We perform massively parallel experimental evolution over up to 40 d and complement this with a computational model to infer the underlying population-dynamical parameters. We find that in our study, combination therapy outperforms monotherapies, as well as cycling and mixing, in minimizing resistance evolution and maximizing uninfecteds, as long as there is no influx of double resistance into the focal treated community.

## Significance

Antibiotic combination therapy is a promising strategy to combat the rising problem of resistance. However, developing such strategies is hindered by the lack of an experimental system that allows testing of strategies in a realistic epidemiological context. We present an approach to test the effects of different treatment strategies in vitro in order to narrow the gap between computational and clinical studies. Using a laboratory automation system, we approximate the epidemiological population dynamics expected in a hospital setting using experimentally evolving bacterial populations. We show that as long as there is no influx of double resistance into the system, combination therapy outperforms all other tested strategies, even at concentrations lower than those used for monotherapy.

**Fig. 1.**
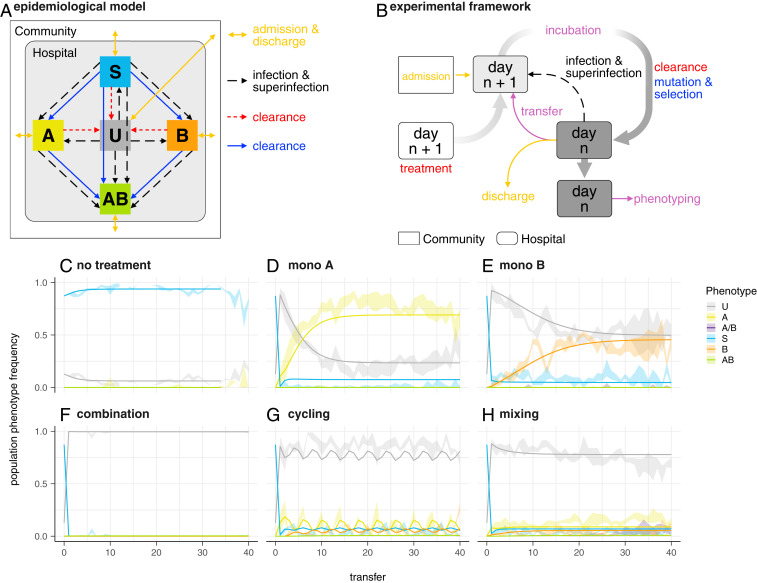
(*A*) Schematic diagram of the dynamical epidemiological model used (*Materials and Methods* and *SI Appendix*). Colors correspond to processes in *B* and phenotypes in *C–H*. (*B*) Schematic representation of one treatment arm in the experimental framework. Rounded rectangles represent a hospital (microtiter plate) containing uninfected (wells containing only bacterial growth medium) and infected (growth medium with sensitive or resistant strains) patients. Plates for day *n* + 1 are prepared with medium and antibiotics of the corresponding treatment arm and are then inoculated from the community (rectangle) and/or from the previous day’s plate according to the chosen scenario (see *Results*). Infection and superinfection are modeled by additional transfers from the previous day’s (*n*) plate. After incubation, cultures are used for the next day’s inoculation, and all cultures are spotted on agar plates for the determination of population phenotypes. Colors correspond for processes in *A*; purple is used for procedures with no direct equivalent in the epidemiological model. (*C*–*H*) Population phenotype frequency during experimental evolution in the absence of preexisting resistance (scenario ∅). Ribbons indicate the observed range in four replicate populations. Lines denote the frequency of population phenotypes after each transfer based on a model fit to all data simultaneously using the mean of the posterior distribution for each parameter. A, resistant to nalidixic acid; B, resistant to streptomycin, A/B, mixed population of single resistants; AB, resistant to both drugs; S, sensitive; U, uninfected patients. (*C*) No treatment. The gap at transfer 13 is due to missing data resulting from a mechanical malfunction of the setup. (*D*) Monotherapy A (nalidixic acid). (*E*) Monotherapy B (streptomycin). (*F*) Combination therapy. (*G*) Cycling: Treatment is switched every two transfers between nalidixic acid and streptomycin. (*H*) Mixing: Treatment with nalidixic acid or streptomycin is randomly assigned to each well for each transfer. In all treatment strategies, each antibiotic is used at 2× MIC.

Modern medicine critically relies on the availability of potent antibiotics. The rapid rise of resistance, together with the slowing rate of discovery of new antibiotics, threatens to undermine future options of antibiotic therapy (1). To preserve the efficacy of currently available antibiotics, we need to find approaches for a more sustainable use. Clearly, reducing unnecessary and inappropriate use of antimicrobials is a public health priority (2). In many situations, however, treatment is required, which raises the question of how to best treat with antibiotics while simultaneously minimizing the risk of antibiotic resistance. For the treatment of pathogens with high potential to develop resistance, such as HIV, *Mycobacterium tuberculosis*, or *Plasmodium falciparum*, combination therapy has been highly successful in reducing treatment failure due to resistance and has become the standard of practice (3). For hospital-acquired bacterial infections, combination therapy is less common. Other multidrug treatment strategies, such as cycling or mixing, have been investigated quite extensively by using computational models (4–14) and, to a lesser extent, by randomized clinical trials (RCTs) (15–17). Computational models allow one to explore a broad range of treatment strategies and often show that combination therapy can be beneficial, but are naturally limited in their realism. Clinical studies, however, often do not show superiority of combination therapy, but allow one to study only a limited set of scenarios. What impedes progress in the field is the lack of a practicable framework for experimental epidemiology, which allows testing of the effect of treatment scenarios under realistic epidemiological population dynamics. Animal models, however, are impractical due to the difficulty of controlling experimental conditions, the prohibitive costs, and the limited ability to compare epidemiological spread to humans. As a possible alternative, we present here an approach to compare the effect of alternative treatment strategies using a robotic liquid-handling platform. With this platform, we study resistance evolution in vitro under treatment implementing realistic epidemiological population dynamics for infection and patient migration. We complement this by simulating the same processes using an epidemiological model (Fig. 1*A*).

Our approach mimics a hospital with continuous admission and discharge of patients (Fig. 1*B*). The central unit representing an individual patient is a well in a microtiter plate, which can either contain only bacterial growth medium (uninfected patient/well), or contain sensitive or resistant strains (infected patient/well). As a model organism, we used *Escherichia coli* MG1655. To increase the likelihood to observe de novo resistance, we used a *mutS* knockout which exhibits an around 100-fold higher mutation rate than the wild type.

Treatment was simulated by adding antibiotics to the wells (treated patient/well). We used two antibiotics, streptomycin (A) and nalidixic acid (B). These antibiotics were chosen because they have different modes of action (protein synthesis inhibition and gyrase inhibition, respectively) and because for each antibiotic, there are well-described single point mutations that show no epistasis (18), and strains of *E. coli* adapted to these drugs did not show collateral resistance (19). Furthermore, at subinhibitory concentrations, these two antibiotics do not show synergistic or antagonistic interactions and show similar, bactericidal, time-kill kinetics (20). While nalidixic acid is no longer in clinical use, newer quinolones (such as ciprofloxacin) with the same mode of action are still widely used. Streptomycin is widely used and is considered a critically important antibiotic, according to the World Health Organization (21).

We considered three different combination strategies: combination therapy, where patients are treated with both antibiotics simultaneously; cycling, meaning temporal rotation of treatment with either antibiotic; and mixing, meaning the random treatment of half the population with antibiotic A and the other half with antibiotic B at the same time. In addition, we considered the two monotherapy strategies with either antibiotic alone, and we also included no treatment as a control.

Each treatment strategy was implemented in four replicates on a 384-well plate, thus simulating four replicate hospitals, each with 94 patients (plus eight control wells to monitor unintended cross-contamination). Bacterial populations were grown for 24 h at 37 °C, representing 1 d in the hospital. Then, a fraction of wells on the incubated plate were chosen randomly to represent turnover due to discharge and admission of patients. The average stay of a patient was thus independent of the disease status. From all other wells, a small volume was transferred to new microtiter plates containing growth medium and antibiotics corresponding to the treatment regimen. For all treatment regimens, including combination therapy, each antibiotic was used at twice the minimal inhibitory concentration (MIC). We chose a concentration above the MIC, but low enough to allow for the emergence of de novo resistance in our relatively small populations (approximately $10^{7}$ colony-forming units [CFU]/mL). At these concentrations, we observed similar resistant mutant frequencies for the sensitive strain used in these experiments (*SI Appendix*, Fig. S2*A*). Mutation probabilities to high-level resistance were different for nalidixic acid and streptomycin, but were the same irrespective of existing resistance to the other drug (*SI Appendix*, Fig. S2*B*).

The wells chosen to represent patient discharge and admission were not transferred, but instead received a small inoculum from stock populations representative of the prevalence of uninfected, sensitive-, and resistant-infected individuals in the community outside the hospital. Infection and superinfection were simulated by transferring small volumes from one well to another. This infection step was identical across all treatment arms, but the probability of transmission depended on the effect of the treatment on bacterial densities. The above procedure was iterated for many days to simulate patient dynamics, as may be observed in hospitals, and the effects of alternative treatment regimens on the evolution and spread of antibiotic resistance were compared. For better readability, we will refer to the wells as patients when referring to the epidemiological level of the population dynamics. For further details, see *Materials and Methods*.

## Results

### Combination Therapy Outperforms Other Strategies.

We compare alternative treatment regimens based on their effect on 1) maximizing the frequency of uninfected individuals and 2) minimizing the frequency of individuals infected by a resistant strain. The first criterion can be applied to all six treatment arms. The second one, however, does not allow comparison with control using no antibiotics, as the control trivially prevents the evolution of resistance. The resistance phenotype of the bacterial population in a well was measured by transferring small aliquots from the well onto four different agar plates containing either no antibiotic, only antibiotic A or B, or both. An A-resistant phenotype would thus grow on the no-drug and the A-containing plate, but not on the B- and AB-containing plates. Additionally, we assessed resistance phenotype by growth (i.e., optical density at 595 nm [*OD*_595_] > 0.1) in the experimental cultures.

For the first scenario (scenario ∅), we assumed that there was no influx of antibiotic-resistant infections from outside (i.e., the wells representing newly admitted patients contained either no bacteria or sensitive bacteria only). Hence, all resistance observed evolved in response to treatment in the hospital. Patients were discharged and admitted with a rate of 0.2 per day. The rate of infection was 0.3. The time course of 40 successive days of treatment for all six treatment arms is shown in Fig. 1 *C*–*H*. With regard to the treatment effect on the frequency of uninfecteds, we found that combination therapy was most effective, as the entire infected population was cleared throughout the course of the experiment. Cycling and mixing lead to a lower level of uninfecteds, but still substantially higher than both monotherapies. Because of the strong bottleneck at every transfer (a dilution of 1/166), low-frequency, nongrowing phenotypes, such as persisters, are not expected to be maintained in the experiment. With regard to the treatment effect on resistance, again, combination therapy outperformed the other strategies. The resistance frequency under cycling and mixing was similar, and lower than under the monotherapies. Surprisingly, resistance to both antibiotics did not emerge and spread, despite the fact that there was at least one combination of two single-point mutations (i.e., *gyrA* S83 L and *rpsL* K43 R), each giving high-level resistance to one antibiotic, that conferred high-level resistance to both antibiotics at low fitness cost (18). Sequencing the resistance-determinant regions of *gyrA* and *rpsL* revealed mostly different mutations at those positions (*SI Appendix*, Table S2). Trindade et al. (18) report both positive and negative epistatic interactions between mutations at these positions. This raises the possibility that these interactions prevent the evolution of double resistance in our experiment.

The findings, that combination therapy outperforms the other strategies, and that cycling and mixing have similar effects, are in good agreement with the computer simulations of Tepekule et al. (12). Moreover, the observation that cycling and mixing have similar effects is also in agreement with the outcomes of a recent multicenter RCT (17).

### Parameter Estimates Vary across Different Treatment Arms.

**Fig. 2.**
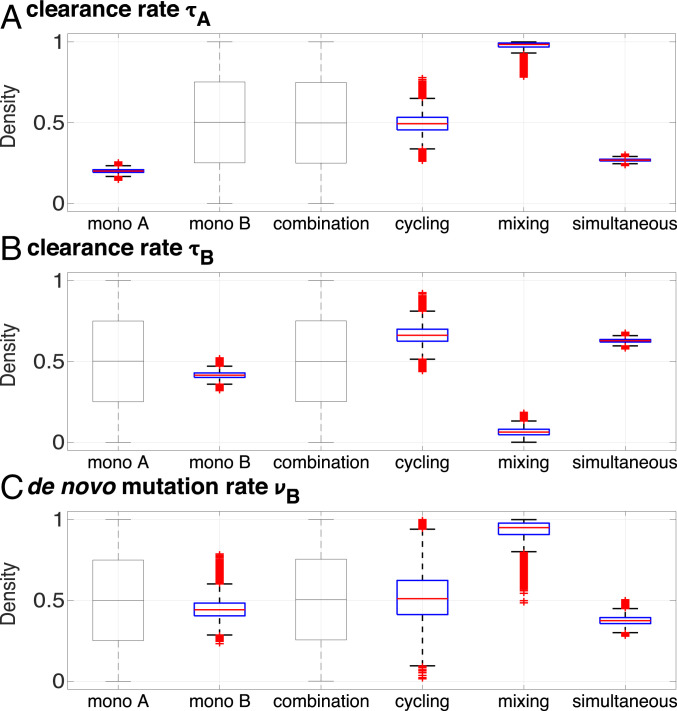
Posterior distributions (estimated probability distribution for a given parameter [*Materials and Methods* ]) obtained from fitting the model to different parts of the experimental data. Mono A, mono B, combination, cycling, and mixing refer to fitting the model to data only from these treatment arms, whereas simultaneous refers to the overall fit to the time course of all treatment arms simultaneously. *A*, *B*, and *C* show, respectively, the posterior distributions of the parameters $\tau_{A}$, $\tau_{B}$, and $\nu_{B}$ describing the clearance rate after treatment with antibiotic A, clearance rate after treatment with antibiotic B, and the rate of de novo emergence of B resistance for the sensitive strain. Posterior distributions of the parameters that cannot be identified from a given treatment arm are shown as thin box plots and equal to their prior distribution, which is uniform between zero and one. Posterior distributions, including all model parameters, are presented in *SI Appendix*, Fig. S5.

Fig. 1 *C*–*H* also shows the population dynamics of a model that is fitted simultaneously to the time course of all treatment arms. The model is an extension of the model used by Tepekule et al. (12) (*Materials and Methods* and *SI Appendix*). Fitting the model independently to each treatment improved the fit by permitting the same parameter to have different posterior distributions for different treatment arms. Ideally, we would expect similar posterior distributions for a given parameter across those treatment arms for which the parameter can be estimated, since building an epidemiological model fundamentally assumes that the dynamics of different treatment arms can be explained by using the same set of processes. The identifiability of the parameters for the different treatment arms is shown in a Venn diagram (*SI Appendix*, Fig. S4). However, fitting the model separately to each treatment revealed substantial differences for some key parameters between the alternative treatment regimens (Fig. 2). Although the same processes (e.g., de novo evolution of resistance in response to drugs) occurred in the monotherapies and cycling or mixing, parameter estimates obtained from the different treatment arms differed substantially. This implies that the established approach of using a general model succeeds in explaining the dynamics of different treatment outcomes up to a certain level (Fig. 1 *A*–*F*), but does represent an oversimplification by excluding treatment-specific processes.

### Cycling and Mixing Perform Better than Predicted Based on Monotherapies.

Although the model gives an overall good fit to all treatment arms, it systematically overestimates the frequency of uninfecteds in monotherapy A and underestimates it for cycling. The differences in key parameters (Fig. 2) indicate that the population dynamics of multidrug strategies, such as cycling and mixing, cannot be predicted accurately from measurements based on the monotherapies only. Importantly, using the parameter estimates from the monotherapies to model the outcome of cycling and mixing leads to an overestimation of the frequency of resistants and an underestimation of the frequency of infecteds, implying that both cycling and mixing perform better than would be anticipated on the basis of the effect of the monotherapies (*SI Appendix*, Fig. S3).

### Preexisting Double Resistance Negates Benefit of Combination Therapy.

**Fig. 3.**
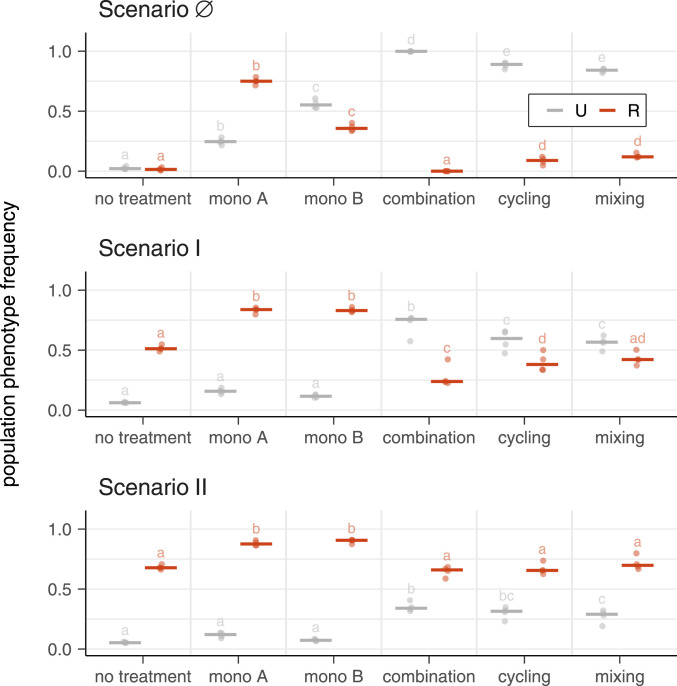
Frequencies of uninfected and resistant populations for different treatment strategies in different resistance inflow scenarios. Points show population phenotype frequency averaged over transfers 9 to 12 for four biological replicates for scenarios ∅ (no inflow of resistance), I (inflow of both single resistances), and II (inflow of double resistance). Bars show the median. Samples not sharing a letter are significantly different (generalized linear hypothesis test across treatment strategies, *P* < 0.05; ANOVA tables and all *P* values can be found in *SI Appendix*, Tables S4–S9). R, resistant populations; U, uninfected (sum of phenotypes A, B, A/B, and AB; Fig. 1). The full time series are shown in *SI Appendix*, Figs. S6–S8.

For scenario ∅ (admission of uninfected and sensitive infected patients only), combination therapy outperforms all other treatment strategies. But how does combination therapy perform when resistant patients are also admitted? To address this, we studied two further scenarios with progressive levels of resistant inflow into the hospital. In scenario I, also, patients were admitted who carry bacteria resistant to a single antibiotic. In scenario II, additionally, patients carrying double resistance were admitted. For the three scenarios, the different treatment regimens were compared on the basis of the frequencies of uninfecteds and resistants, respectively, over transfers 9 to 12 (Fig. 3; full time series are shown in *SI Appendix*, Figs. S7–S8). Under scenarios ∅ and I, combination therapy significantly outperformed the other treatment regimens, with regard to both maximizing the frequency of uninfecteds and minimizing the frequency of resistants. In scenario II, combination therapy was no longer significantly different from cycling and mixing, and all strategies selected for high levels of double resistance. In summary, combination therapy offers a benefit, as long as there is no influx of double resistance, and all strategies fail otherwise.

### Benefits of Combination Therapy Do Not Arise only from Higher Dosage.

**Fig. 4.**
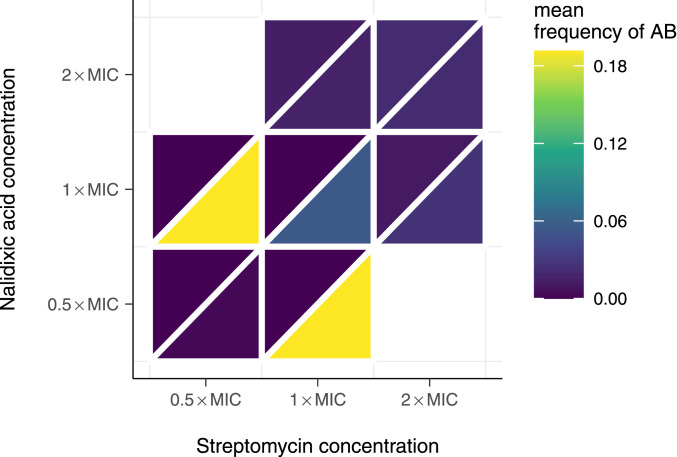
Mean frequencies of double-resistant populations after five transfers at different concentrations of nalidixic acid and streptomycin. The upper left triangle indicates frequency of double resistance to a high concentration of drugs used on plate (10× MIC), while the lower right triangle indicates double resistance to the drug concentrations used in liquid culture. Full time series of population phenotype frequencies can be found in *SI Appendix*, Fig. S9.

Does the advantage of combination therapy stem from increased drug dosage? More specifically, does combination therapy outperform the other strategies because it is based on using each antibiotic at the respective concentration used in the single drug therapies? To address this question, we performed another series of analogous experiments for combination therapy, lowering the drug concentration from 2× MIC, as used in scenarios ∅, I, and II, to 1× and 0.5× MIC for both drugs (plus a control of no drugs). We measured the effect on the emergence of double resistance in two ways: 1) growth on solid medium containing 10× MIC, indicating high-level resistance; and 2) growth in liquid culture at the respective drug concentration used for treatment indicating low-level resistance. The results show that combination therapy prevents the emergence of both high- and low-level double resistance, as long as the concentration of both antibiotics are 1× MIC or higher (upper left triangles, Fig. 4). This finding argues that it is not the effect of drug dosage, but the probability of simultaneous acquisition of multiple mutations that underlies the success of combination therapy. For lower concentrations, low-level, but not high-level, resistance evolves (lower right triangles, Fig. 4). Note, however, that the concentrations used in liquid are below MIC for at least one antibiotic, where even bacteria sensitive to these antibiotics are expected to grow. Given that at 1× MIC for both antibiotics, combination therapy prevents the emergence of resistance; this indicates that the benefit of combination therapy does not arise only from the higher combined concentration compared to the other treatment strategies. This is another possible benefit of combination therapy: Combining antibiotics might enable the reduction of the concentration of potentially toxic antibiotics, while still minimizing the likelihood of resistance evolution.

## Discussion

In summary, we find that, as long as there is no influx of double resistance into the focal population, combination therapy outperforms all other strategies considered here, both in terms of maximizing frequency of uninfecteds and minimizing the frequency of resistants. Note that the superiority of combination therapy is based on criteria that focus only on the evolution of resistance and the number of uninfecteds, but disregard other aspects that are highly relevant in clinical practice, such as side effects. The advantage of combination therapy, however, does not appear to result from the higher total antibiotic concentration when combining antibiotics. When there is influx of double resistance, all strategies fail, as they all result in the increase of double resistance. In other words, combination therapy is best at preventing the evolution of resistance, and all strategies are poor at managing preexisting resistance.

The findings are in full agreement with theoretical predictions (4, 12). Moreover, the finding that cycling and mixing have similar effects is supported by a recent multicenter RCT (17). The observed superiority of combination therapy, however, is somewhat in contradiction with empirical observations. Two meta-analyses of RCTs (15, 16) compared monotherapy with a single β-lactam to combination therapy with (typically a different) β-lactam and an aminoglycoside. These studies concluded that combination therapy does not offer an advantage over monotherapy with regard to the emergence of antibiotic resistance. While combination therapy did reduce treatment failure due to the emergence of resistance (15), monotherapy was better or noninferior with regard to other clinical endpoints, such as emergence of resistance, mortality (either all-cause or attributable to infection), or superinfection. In general, RCTs comparing combination therapy and monotherapy are scarce, in particular with regard to resistance as a primary clinical endpoint (15). Given the success of combination therapy in reducing the emergence of resistance in other infections such as HIV, malaria, or tuberculosis, in our view, combination therapy deserves further investigation as a strategy to prevent antibiotic resistance also for nosocomial infections.

We were surprised by the lack of double resistance observed in our experiments, given previous reports indicating that positive epistasis can drive the evolution of double resistance for the drugs used (18). The resistant strains used for scenarios I and II are based on single point mutations that confer high-level resistance to each drug. When combined, they also confer high-level double resistance at low cost (18). Sequencing of populations selected from both monotherapies, as well as the cycling and mixing treatments, revealed mutations at previously described locations in both *gyrA* and *rpsL* (18) (*SI Appendix*, Table S2). However, the precise mutations that we introduced into our strains were found rarely. Trindade et al. (18) do not only find positive epistasis, but also mutations that seemed to be lethal when combined. Although some of the mutations that we found are different from those studied by Trindade et al. (18), a possible explanation for the absence of double resistance could be strong negative interactions between resistance mutations evolving in our experiments.

The presented results corroborate the feasibility of our approach to study resistance evolution using a framework based on in vitro epidemiology. We demonstrate that massively parallel long-term experimental evolution of antibiotic resistance under realistic epidemiological population dynamics is possible in an automated setup. We have investigated here a limited set of scenarios. Importantly, we only consider chromosomal mutations that are not horizontally transferred. However, while plasmid-mediated resistance is a big concern, chromosomal resistance mutations also contribute to resistance in many important pathogens (22, 23).

It is important to state that to what extent the behavior of our experimental model would fit the results of the epidemiological model was not known a priori. Fundamentally, the same processes, i.e., mutation and selection, are happening in all treatment arms. However, while our experimental model qualitatively captures the behavior, there are quantitative differences, especially with regard to the cycling and mixing strategies. The discrepancy among the posterior distributions obtained from different treatment arms highlights that the population dynamics in a given treatment arm cannot be fully captured by using the estimates of the identifiable parameters from the other treatment arms.

Our model does not consider stochastic effects, which is a limitation when modeling extinction events. Although, in theory, this might affect the frequency of the resistant strains, especially for multidrug therapies, we believe that our estimation results would be robust to such stochastic effects, since we have not observed any extinction events in the absence of treatment.

Future studies could increase realism by studying the effect of treatment strategies when resistance is plasmid-borne or when compliance to the prescribed regimen is imperfect, which both could affect the superiority of combination therapy. Furthermore, in vitro studies allow the consideration of a much broader array of drugs and bacterial strains, as effects of combination therapy are dependent on both the strain and the drug (20, 24, 25). Given the impracticalities of animal models for experimental epidemiology, our approach may present a valid attempt to narrow the gap between computational and clinical studies on antibiotic-resistance evolution.

## Materials and Methods

### Strains, Drugs, and Media.

All strains were grown in minimal salts (MS) medium [MS contains 1 g/L (NH_4_)_2_SO_4_, 3 g/L KH_2_PO_4_, and 7 g/L K_2_HPO_4_ supplemented with 0.4 mM MgSO_4_, 3.33 nM FeSO_4_, 1.2 mM Na_3_C_6_H_5_O_7_, and 0.8 ng/L thiamine], and 0.2% glucose was added as a carbon source. Furthermore, 15 μg/mL chloramphenicol was added to maintain the used mutants and to prevent external contaminations. Solid medium was prepared the same way, but supplemented with 1.5% (weight/volume) agar.

Streptomycin was used at a concentration of 12.5 or 100 μg/mL in liquid and solid medium, respectively. Nalidixic acid was used at a concentration of 20 or 40 μg/mL in liquid and solid medium, respectively. These concentrations correspond to 2× MIC in liquid and 10× MIC (as measured by using a standard broth microdilution assay) on solid medium for both antibiotics.

All strains used were derived from *E. coli* K-12 MG1655. A *gyrA* S83 L (C248 T) or a *rpsL* K43 R (A129 C) mutation was added by using single-stranded DNA recombineering (26) and subsequently transduced alone or in combination into a fresh background by using P1 transduction (27, 28). The mutations conferred resistance to nalidixic acid or resistance to streptomycin, respectively, at relatively low fitness cost (18) and were selected on plates containing 40 μg/mL nalidixic acid and/or 100 μg/mL streptomycin. Additionally, and following the same methodology, a fluorescent marker linked to a chloramphenicol resistance gene (*cat*-YFP) from *E. coli* MDS42(YFP) (29) was transduced into the single- and double-resistant strains and selected on plates containing 25 μg/mL chloramphenicol. In order to increase mutation supply, the *mutS* knockout from *E. coli* JW2703 (30) was also transduced into the strains and selected on plates containing 25 μg/mL kanamycin, resulting in the strains used in the experiment. All used genotypes were verified by resequencing of the modified genes. A list of all strains and their genotype can be found in *SI Appendix*, Table S3.

### Evolution Experiments.

In order to test the effect of different treatment strategies on the evolution of antibiotic resistance, we set up large-scale, serial passage evolution experiments using a Tecan Evo 200 automated liquid handling system (Tecan) with an integrated, automated incubator (Liconic STX100, Liconic) and a Tecan Infinite F200 spectrophotometer (Tecan).

Our experiments were designed to simulate a hospital or hospital ward. We modeled beds as wells in a 384-well plate (Greiner catalog no. 781186) and patients as growth media in those wells. All beds were always occupied, i.e., filled with a total of 50 μL of growth medium, allowing for a maximum population size of about $10^{7}$ CFU. One 384-well plate contained four replicate units with 94 patients each. The remaining eight wells were used as sterile controls. We serially passaged every well every day into new plates, incorporating the following processes: 1) treatment; 2) admission and discharge; 3) transfer; 4) infection and superinfection; and 5) phenotyping. Before each transfer, a custom R script computed the necessary pipetting steps using the parameters outlined below and prepared pipetting worklists. For scenario ∅, we initially planned the experimental evolution experiment for 40 transfers. However, we decided to extend the experiment and switch the two monotherapies and to continue the experiment for another 12 transfers. We finished the experiment after 52 transfers; however, we here focus on the 40 transfers before the drug switch. The complete time series for scenario ∅ can be found in *SI Appendix*, Fig. S6. Scenario I and scenario II were run for 13 and 22 transfers, respectively. Because these experiments were largely dominated by preexisting resistance, and because of the trajectories observed in the scenario ∅, we decided to stop these scenarios earlier, as the dynamics had already run its course (*SI Appendix*, Figs. S7 and S8). Every 10 transfers, and at the end of each experiment, 35 μL of 40% glycerol was added to the assay plates, and the plates were stored at −80 °C.

### Treatment.

First, we prepared new 384-well plates by filling all wells with 40 μL of growth medium. We then added 5 μL of diluted antibiotic stock solution (20× final concentration, or no antibiotic for control wells and wells without treatment) to all wells, according to the treatment strategy. We used a separate 384-well plate for every treatment strategy. All wells, except the sterile controls, were treated, irrespective of their infection status. We considered the following treatment strategies: 1) no treatment; 2) monotherapy with nalidixic acid; 3) monotherapy with streptomycin; 4) combination therapy with both drugs; 5) cycling, where the used antibiotic was changed every other transfer; and 6) mixing, where one of the antibiotics was randomly assigned to each patient. For the initial setup of the first 384-well plate, no antibiotics were added. While all plates were prepared and treated at the same time, the following steps were conducted in series to minimize the difference in incubation time between the plates.

### Admission and Discharge.

The experimental design also reflected the influx of patients from a community outside the hospital. This community was assumed sufficiently large, such that it did not change during the course of the experiment. Experimentally, this was implemented as follows. A certain fraction of wells (0.2 for all experiments reported here) were randomly chosen for admission and discharge. These wells were exempt from the transfer to the new plate (see *Transfer* section). Instead, these wells were inoculated with 5 μL from replicate overnight cultures, or sterile medium for input of uninfecteds, that was prepared from frozen stocks every day for the following day. This represents patient influx from the outside community. The fraction of the different phenotypes (uninfecteds, sensitive infecteds, single-resistant A, single-resistant B, and double-resistant AB) constituting the overall influx differed for the different scenarios considered. These proportions were (0.15,0.85,0,0,0) for scenario ∅, (0.21,0.57,0.11,0.11,0) for scenario I, and (0.21,0.52,0.11,0.11,0.05) for scenario II, respectively. All wells that were not chosen received 5 μL of sterile medium to equalize culture volumes.

### Transfer.

Next, all wells that had not been chosen for admission and discharge were diluted into the new plate at the same position. This was done by using a custom pintool developed in-house that allows excluding certain positions by removing certain pins (i.e., the pins corresponding to the wells that are chosen for admission and discharge). The pintool transferred a fixed volume of about 0.3 μL of the culture for a dilution of approximately 1/166 at each transfer (about 5 × 10^4^ CFU). For the initial setup, this step was skipped.

### Infection and Superinfection.

To implement infection, we randomly chose a fraction of wells (0.3 in all experiments) as the sources of infection. The targets of infection were chosen randomly with replacement from the remaining wells. Approximately 0.3 μL of the chosen source wells were then transferred from the previous plate to the chosen target well on the new plate by using the custom pintool for a dilution of approximately 1/166 (approx. 5 × 10^4^ CFU). The newly prepared plates were then moved to the incubator and incubated at 37° C and 95% relative humidity for approximately 24 h.

### Phenotyping.

After the newly prepared plates were placed in the incubator, an aliquot of the cultures from the previous transfer was spotted onto agar plates containing no antibiotic, nalidixic acid, streptomycin, or both antibiotics. The agar plates were then also moved into the incubator. At the beginning of the next transfer, agar plates were fetched from the incubator, and a picture of each plate was taken. Pictures were analyzed for growth (growth/no growth) by using the Pickolo Software package (SciRobotics), as well as manual inspection of each picture by using a custom R script.

To measure growth of cultures in the 384-well plates, *OD*_595_ of each well was measured at the beginning of each transfer. We defined growth in 384-well plates as reaching an $OD_{595}>0.1$ after incubation.

From these two measurements, we defined six possible phenotypes: *U* (uninfected), for populations that showed no growth on any of the agar plates and in liquid culture; *S*, (sensitive) for populations that grew only in the absence of antibiotics on agar or in liquid culture; *A* or *B*, for populations that grew on agar plates or in liquid culture in the presence of nalidixic acid or streptomycin, respectively; *A/B*, for populations that grew in the presence of nalidixic acid and streptomycin (on agar plates or in liquid culture), but not in the presence of both drugs at the same time, indicating a mixed population; and *AB*, for populations that grew both in the presence of both single drugs and the combination. All other populations were classified as *E*, for erroneous phenotypes.

### Sequencing.

To identify common resistance mutations, we picked three random populations from time points between 20 and 40 from all four replicates of the monotherapy treatments, as well as cycling and mixing. Populations were inoculated from frozen samples and grown for 24 to 48 h, and DNA was isolated by using the Wizard Genomic DNA purification kit (Promega). We sequenced the main resistance-determinant regions for streptomycin and nalidixic acid, which are in *gyrA* and *rpsL*, respectively (18). The regions were amplified and sequenced by using the same primers as used in Trindade et al. (18).

### Statistical Analysis.

To compare the three different scenarios studied, we averaged the population phenotype frequency over transfers 9 to 12 for all four biological replicates for each scenario. Transfers 9 to 12 were chosen because they are the latest timepoints for which we have data for all three scenarios. The effect of different treatment strategies on the frequencies of uninfected and resistant populations in the three different scenarios was tested by using an ANOVA, followed by a post hoc generalized linear hypothesis test for a model that included both treatment strategies and phenotype as a main effect, as well as their interaction. ANOVA tables, as well as all tested linear hypotheses and the corresponding test statistics, can be found in *SI Appendix*, Tables S4–S9. All statistical analyses were performed in R 4.0.2 (31) using the packages tidyverse (32), multcomp (33), and multcompView (34).

### Mathematical Modeling.

Adapting the mathematical model by Tepekule et al. (12), we used a compartmental model to describe the population dynamics observed in the evolution experiment. The mathematical model is described in greater detail in *SI Appendix*. The model considered each well as a patient and ignored the population dynamics of bacteria within the wells. Hence, at each time point, each well belonged to one of the five compartments: *U*, *S*, *A*, *B*, or *AB*. The phenotype *A/B*, which represents a mixed population of single resistants, was not considered in the model, since the frequency of *A/B* in the experiment was negligible compared to the other phenotypes. The epidemiological processes included in our mathematical model are analogous to those implemented during the experiments, which were admission and discharge of the patients, infection, superinfection, clearance due to successful treatment, and de novo emergence of resistance during incubation (Fig. 1*A*). Each process was associated with a rate, and these rates were translated into model parameters of the corresponding system of ordinary differential equations (ODEs). The system of ODEs and the model parameters with their corresponding descriptions and units are provided in *SI Appendix*.

### Parameter Estimation and Model Fitting.

We estimated the parameters of the ODE-based model by fitting it to the time course of the frequency of the different phenotypes observed for scenario ∅, as this experiment had the largest number of successive days of treatment. Since our experimental results are highly replicable (Fig. 1 *C*–*H*), we used the mean frequency of phenotypes averaged over the four replicates. To fit the mathematical model to the experimental data, we implemented the Metropolis–Hastings algorithm (35) and used the least-squares method (36), assuming that the error between the model outcomes and the experimental data were normally distributed (see also *SI Appendix*). By minimizing this error, we obtained posterior distributions for each parameter, which were then used to simulate the mathematical model. A uniform prior distribution on the interval (0,1) (U(0,1)) was assumed for all parameter values. We implemented two different fitting procedures, where 1) the parameters were estimated separately for each treatment by minimizing the error independently for different treatment arms (independent estimation); and 2) the parameters were estimated simultaneously by minimizing the error over the concatenated time course of all treatments (simultaneous estimation). Posterior distributions of the model parameters for independent and simultaneous estimations are given in *SI Appendix*, Fig. S5.

## Supplementary Material

Supplementary File

## Data Availability

Experimental data (37) and analysis scripts, as well as code for the mathematical model and parameter estimation data (38), have been deposited in Zenodo (https://doi.org/10.5281/zenodo.4537380 and https://doi.org/10.5281/zenodo.3819350).
