# Supplementary material for: Comparing treatment strategies to reduce antibiotic resistance in an in vitro epidemiological setting
Source: Proc Natl Acad Sci U S A. 2021 Mar 25;118(13):e2023467118. doi: 10.1073/pnas.2023467118 (PMC8020770; doi:10.1073/pnas.2023467118)
Supplement: Supplementary File [file pnas.2023467118.sapp.pdf]

PNAS
www.pnas.org

Supplementary Information for

Comparing treatment strategies to reduce antibiotic resistance in an *in vitro* epidemiological setting

Daniel C. Angst, Burcu Tepekule, Lei Sun, Balázs Bogos, Sebastian Bonhoeffer

Corresponding Author: Daniel C. Angst
E-mail: daniel.angst@env.ethz.ch

This PDF file includes:

Supplementary text
Figs. S1 to S9
Tables S1 to S9
SI References

Supporting Information Text

Mathematical model

The population dynamics outlined in Fig. 1a in the main text are reflected by the system of ordinary differential equations (ODEs) given by Eqns. 1–5. The variables U, S, A, B and AB reflect the populations of uninfecteds, sensitive infecteds, and resistant infecteds to drugs A, B, or both, as described in the main text. The descriptions of the parameters of the model are given in Table S1.

$$\begin{aligned}
\frac{\partial U}{\partial t} =& +(1-m_0)\mu - \mu U + (\tau_A f_A + \tau_B f_B + \tau_{AB} f_{AB}) m_0 \mu && \text{Turnover: U} \to - \\
& \pm 0 && \text{Mutation: } - \to - \\
& +(\tau_A f_A + \tau_B f_B + \tau_{AB} f_{AB}) S && \text{Clearance: S} \to \text{U} \\
& +\tau_B (f_B + f_{AB}) A && \text{Clearance: A} \to \text{U} \\
& +\tau_A (f_A + f_{AB}) B && \text{Clearance: B} \to \text{U} \\
& -\beta(S)(1-(f_A + f_B + f_{AB}))U && \text{Infection: U} \to \text{S} \\
& -\beta(1-c_A) f_A A U && \text{Infection: U} \to \text{A} \\
& -\beta(1-c_B) f_B B U && \text{Infection: U} \to \text{B} \\
& -\beta(1-c_{AB})(f_A + f_B + f_{AB})(AB)U && \text{Infection: U} \to \text{AB} \\
& \pm 0 && \text{Superinfection: } - \to -
\end{aligned} \quad [1]$$

$$\begin{aligned}
\frac{\partial S}{\partial t} =& +[1-(\tau_A f_A + \tau_B f_B + \tau_{AB} f_{AB})] m_0 \mu - \mu S && \text{Turnover: S} \to - \\
& -f_A \nu_A S && \text{Mutation: S} \to \text{A} \\
& -f_B \nu_B S && \text{Mutation: S} \to \text{B} \\
& -f_{AB} \nu_{AB} S && \text{Mutation: S} \to \text{AB} \\
& -(\tau_A f_A + \tau_B f_B + \tau_{AB} f_{AB}) S && \text{Clearance: S} \to \text{U} \\
& +\beta(S)(1-(f_A + f_B + f_{AB}))U && \text{Infection: U} \to \text{S} \\
& -\beta(1-c_A) f_A S A && \text{Superinfection: S} \to \text{A} \\
& -\beta(1-c_B) f_B S B && \text{Superinfection: S} \to \text{B} \\
& -\beta(1-c_{AB})(f_A + f_B + f_{AB}) S(AB) && \text{Superinfection: S} \to \text{AB}
\end{aligned} \quad [2]$$

$$\begin{aligned}
\frac{\partial A}{\partial t} =& -\mu A && \text{Turnover: A} \to - \\
& +f_A \nu_A S && \text{Mutation: S} \to \text{A} \\
& -(f_B + f_{AB}) \nu_{A(AB)} A && \text{Mutation: A} \to \text{AB} \\
& -\tau_B (f_B + f_{AB}) A && \text{Clearance: A} \to \text{U} \\
& +\beta(1-c_A) f_A A U && \text{Infection: U} \to \text{A} \\
& -\beta(1-c_{AB})(f_B + f_{AB}) A(AB) && \text{Superinfection: A} \to \text{AB} \\
& -\beta(1-c_B) f_B A B && \text{Superinfection: A} \to \text{B} \\
& +\beta(1-c_A) f_A B A && \text{Superinfection: B} \to \text{A} \\
& +\beta(1-c_A) f_A S A && \text{Superinfection: S} \to \text{A}
\end{aligned} \quad [3]$$

$$\begin{aligned}
\frac{\partial B}{\partial t} =& -\mu B && \text{Turnover: B} \to - \\
& +f_B \nu_B S && \text{Mutation: S} \to \text{B} \\
& -(f_A + f_{AB}) \nu_{B(AB)} B && \text{Mutation: B} \to \text{AB} \\
& -\tau_A (f_A + f_{AB}) B && \text{Clearance: B} \to \text{U} \\
& +\beta(1-c_B) f_B B U && \text{Infection: U} \to \text{B} \\
& -\beta(1-c_{AB})(f_A + f_{AB}) B(AB) && \text{Superinfection: B} \to \text{AB} \\
& -\beta(1-c_A) f_A B A && \text{Superinfection: B} \to \text{A} \\
& +\beta(1-c_B) f_B A B && \text{Superinfection: A} \to \text{B} \\
& +\beta(1-c_B) f_B S B && \text{Superinfection: S} \to \text{B}
\end{aligned} \quad [4]$$

$$
\begin{aligned}
\frac{\partial AB}{\partial t} =& -\mu AB && \text{Turnover: AB} \rightarrow - \\
&+ f_{AB}\nu_{AB}S && \text{Mutation: S} \rightarrow \text{AB} \\
&+ (f_A + f_{AB})\nu_{B(AB)}B && \text{Mutation: B} \rightarrow \text{AB} \\
&+ (f_B + f_{AB})\nu_{A(AB)}A && \text{Mutation: A} \rightarrow \text{AB} \\
&\pm 0 && \text{Clearance: } - \rightarrow - \\
&+ \beta(1 - c_{AB})(f_A + f_B + f_{AB})(AB)U && \text{Infection: U} \rightarrow \text{AB} \\
&+ \beta(1 - c_{AB})(f_B + f_{AB})A(AB) && \text{Superinfection: A} \rightarrow \text{AB} \\
&+ \beta(1 - c_{AB})(f_A + f_{AB})B(AB) && \text{Superinfection: B} \rightarrow \text{AB} \\
&+ \beta(1 - c_{AB})(f_A + f_B + f_{AB})S(AB) && \text{Superinfection: S} \rightarrow \text{AB}
\end{aligned}
\qquad [5]
$$

Table S1. Model parameters with their corresponding descriptions and units.

Parameter / Variable	Description	Unit
m_0, $(1-m_0)$	Influx fractions : Admission of patients in the states of S and U, respectively.	-
μ	Turnover rate : Loss of patients due to discharge or death, compensated by admission of new patients	transfer^{-1}
β	Transmission rate of infection $\&$ superinfection	transfer^{-1}
f_A, f_B, f_{AB}	Fraction of patients receiving drug A, drug B, or both drugs	-
c_A, c_B, c_{AB}	Fitness costs of resistance to drug A, drug B, or both drugs	-
ν_A	Rate of *de novo* emergence of A resistance (Transition from the state S to A in the presence of only drug A or both drugs)	transfer^{-1}
ν_B	Rate of *de novo* emergence of B resistance (Transition from the state S to B in the presence of only drug B or both drugs)	transfer^{-1}
ν_{AB}	Rate of *de novo* emergence of double resistance (Transition from the state S to AB in the presence of both drugs)	transfer^{-1}
$\nu_{B(AB)}$	Rate of *de novo* emergence of A resistance given the patient is already resistant to drug B (Transition from the state B to AB in the presence of only drug A or both drugs)	transfer^{-1}
$\nu_{A(AB)}$	Rate of *de novo* emergence of B resistance given the patient is already resistant to drug A (Transition from the state A to AB in the presence of only drug B or both drugs)	transfer^{-1}
τ_A	Rate of recovery in the presence of drug A (Transition from the state S or B to U)	transfer^{-1}
τ_B	Rate of recovery in the presence of drug B (Transition from the state S or A to U)	transfer^{-1}
τ_{AB}	Rate of recovery in the presence of both drugs simultaneously (Transition from the state S to U)	transfer^{-1}

Model Fitting and Parameter estimation

We used two different parameter estimation procedures for model fitting. The first procedure is referred to as independent estimation, where the fitting is done independently for each dataset coming from each treatment strategy. The second procedure is referred to as simultaneous estimation, where combinations of datasets of different treatment strategies are used simultaneously for fitting.

Interpreting the time series data requires two types of models: a process model which is given by Eqns. 1–5, and a statistical model for the deviations between the process model and the data. The statistical model is necessary to evaluate the goodness of the fit of the process model, and thus determine what the optimal parameter values are. Following a well established tradition in model fitting, we use the least-squares method (1), and assume that the deviations between the process model and the data are normally distributed.

The Metropolis-Hastings algorithm is used for each estimation procedure, which is a Monte Carlo Markov Chain (MCMC) method to simulate multivariate distributions (2). A uniform prior distribution on the interval $(0, 1)$ ($\mathcal{U}(0, 1)$) is assumed for all parameter values, since they represent rates with no prior information. The error is assumed to be normally distributed, leading to a likelihood function of

$$\mathcal{L}(\theta|Y) = \prod_{i=1}^{N} f(Y_i|\theta), \qquad [6]$$

$$= \prod_{i=1}^{N} \frac{1}{\sqrt{2\pi\sigma^2}} \exp\left(- \frac{(Y_i - \hat{Y}_i(\theta))^2}{2\sigma^2} \right), \qquad [7]$$

$$= \left(\frac{1}{\sqrt{2\pi\sigma^2}} \right)^N \exp\left(- \sum_{i=1}^{N} \frac{(Y_i - \hat{Y}_i(\theta))^2}{2\sigma^2} \right), \qquad [8]$$

$$= (2\pi\sigma^2)^{-N/2} \exp\left(- \frac{1}{2\sigma^2} \mathrm{SSQ}(\theta; Y) \right), \qquad [9]$$

where θ, Y_i, $\hat{Y}_i$, and σ denote the parameter vector, data point at time i, estimation of the data point at time i, and the standard deviation of error, respectively, and SSQ is the abbreviation for "sum of squares". The natural logarithm of this likelihood is given by

$$\log(\mathcal{L}(\theta|Y)) = -\frac{N}{2} \log(2\pi\sigma^2) - \frac{\mathrm{SSQ}(\theta; Y)}{2\sigma^2}. \qquad [10]$$

Using the Normal likelihood to fit model parameters to data thus requires an estimate for the variance of the deviation between Y and $\hat{Y}$. One can either estimate this from the dataset itself, or use the maximum likelihood estimate of the error variance σ^2, which is equal to $\mathrm{SSQ}(\theta; Y)/N$. Plugging this equality in Eq. 10 leads to

$$\log(\mathcal{L}(\theta|Y)) = -\frac{N}{2} \log \mathrm{SSQ}(\theta; Y) + C, \qquad [11]$$

where C absorbs all the constant terms that are independent of θ, thus can be omitted for optimization purposes. As a result, the final log-likelihood function that is used for the MCMC algorithm becomes

$$\log(\mathcal{L}(\theta|Y)) = -\frac{N}{2} \log \mathrm{SSQ}(\theta; Y). \qquad [12]$$

To calculate the posterior distributions of each parameter, we ran MCMC Metropolis-Hastings algorithm with 10000 iterations per chain for 50 randomly initiated chains. To cover all the possible regions of the parameter space, parameter vectors are randomly initialized at the beginning of each chain. The first half of each chain (first 5000 iterations) is regarded as the burn-in period and discarded. After the posterior distributions for each parameter are estimated for each estimation procedure, median values of these distributions are used as the parameters of the corresponding model, and the least squares estimation $\hat{Y}(\theta)$ is calculated for each treatment strategy separately. Posterior distributions including all model parameters are presented in Fig. S2, and the estimated time series are presented in Fig. 1c–h in the main text in addition to the experimental data.

Next we tested whether the estimated parameters reproduce the experimental finding that combination therapy outperforms the other strategies also in the model. Following the analysis provided in Tepekule et al.(3), we randomly sample parameter space to identify the parameter regions where particular treatment strategies succeed in reducing the number of infecteds the most. Specifically, we sampled from a uniform distribution in the open interval (0,1) for the parameters shown in Fig. S1. Due to the high dimensionality of the parameter space we used linear discriminant analysis (LDA) and projected the results onto the two principal axes of LDA (Fig. S1). We then compared the results of this analysis with the median of the posterior

distributions obtained from the simultaneous estimation, and observed that the set of estimated medians fall into the region where combination therapy outperforms other treatment strategies with a high probability (Fig. S1). The observation that all parameter combinations in the posterior fall into a narrow region that is contained in the region where combination therapy outperforms the other treatment strategies, suggest that superiority of combination therapy is highly robust.

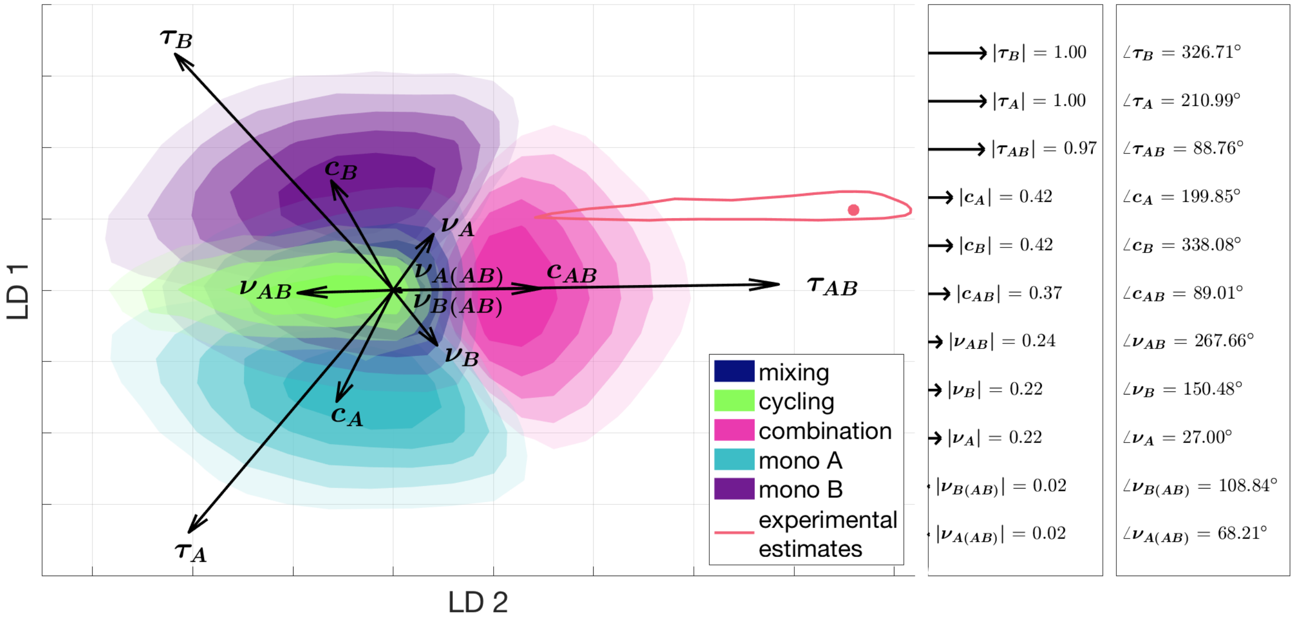

Fig. S1. LDA of random sampling results. LDA classifying parameter sets according to the strategy with the highest gain in the frequency of uninfecteds per transfer. Shaded areas represent the density of the parameter sets coloured according to which strategy wins. Each treatment strategy is represented by a different colour, and the opacity of each colour is proportional to the number of parameter sets that fall into that corresponding region. The red envelope encompasses parameter estimates obtained via simultaneous estimation. 10^6 randomly sampled parameter sets are used. LD1 and LD2 are the two principal axes of the LDA. The parameter vectors are given with their relative magnitudes and counterclockwise angles. The estimated parameters (red envelope) fall into a region where combination therapy is expected to outperform the other strategies

Supplementary Methods

Estimation of mutation probability. Mutation probabilities were estimated using a fluctuation assay (4) following a protocol adapted from Kohanski et al. (5). The fluctuation test was performed in the same medium as used in the evolution experiment (Minimal salts medium with 15µg/m chloramphenicol, see main methods). Briefly, overnight cultures of the sensitive and the two single resistant strain were diluted 1 : 10000 in 50ml medium and incubated for an additional 3.5h at 37°C. Then each culture was diluted 1:3 and ten 1ml aliquots were incubated for 24h at 37°C. All cultures were then plated on solid media without selection as well as on the antibiotics to which the strain is not already resistant to measure mutation probabilities towards those drugs. Plates were counted after 24h incubation at 37°C.

Total colony forming units as well as the distribution of the number of resistant colonies on the different drugs were then used to calculate mutation probabilities using the maximum likelihood method as implemented in the R package `flan` (6) (Fig. S2)

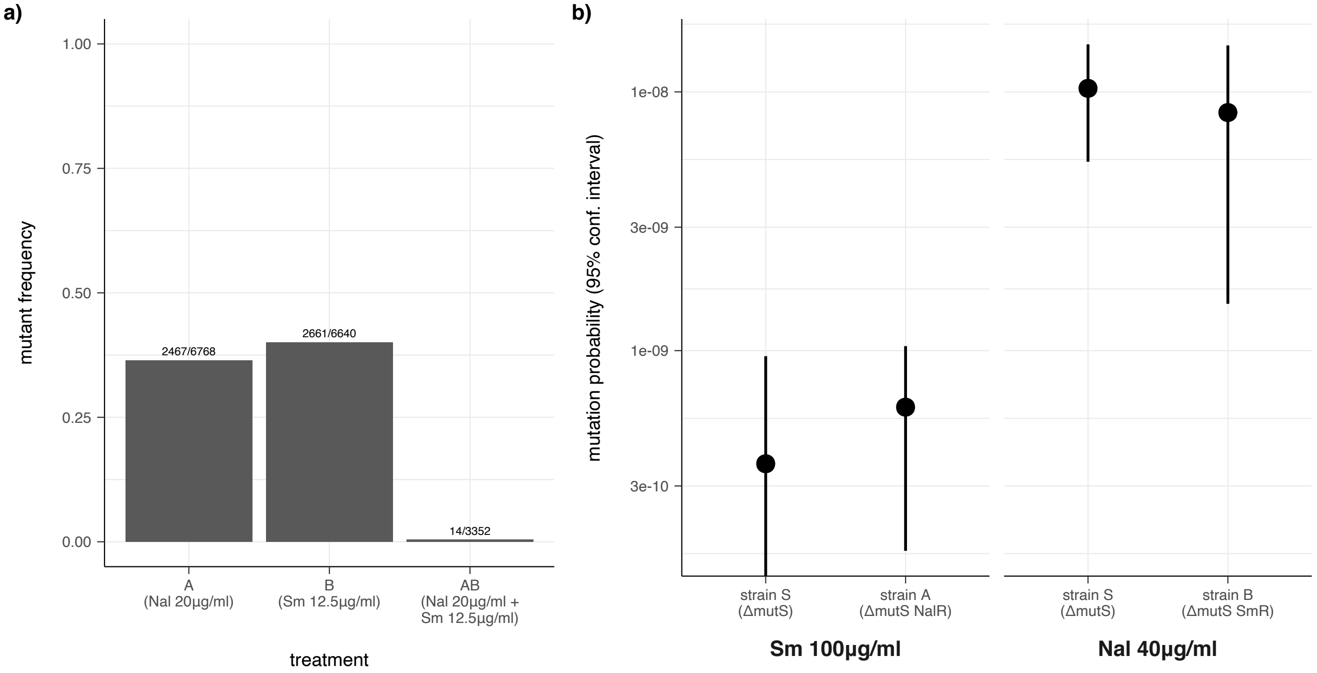

Fig. S2. Mutant frequency in liquid culture and mutation probability as measured in a fluctuation test (a) Frequency of single and double resistant cultures arising from freshly inoculated sensitive cultures in a 52 day evolution experiment (Scenario ∅, see Methods). (b) Mutation probabilities towards 40µg/ml nalidixic acid or 100µg/ml streptomycin for the sensitive and the two single resistant strains used in the experiments. Vertical lines denote the 95% confidence interval.

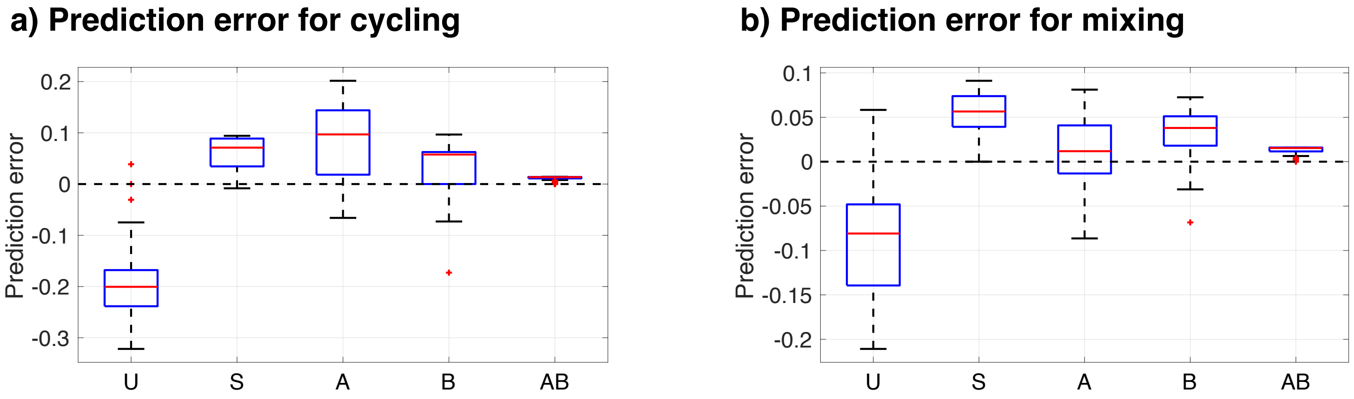

Fig. S3. Distribution of prediction errors for cycling and mixing using parameters estimated via monotherapies. Parameters related to monotherapies are estimated by mono A and mono B, whereas the parameters regarding temporal or spatial alternation of drugs are free to vary in case of cycling and mixing.

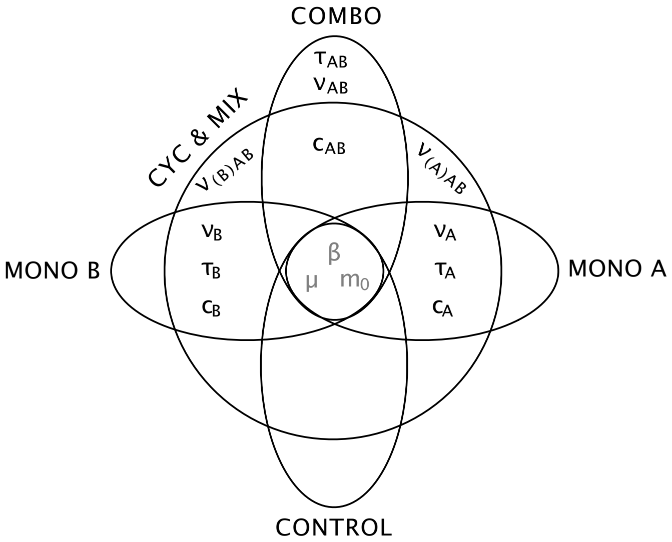

Fig. S4. Venn diagram illustrating the Identifiable parameters for the different treatment arms. Parameters β, and m_0 are colored in gray to indicate that they are not estimated from the experimental data.

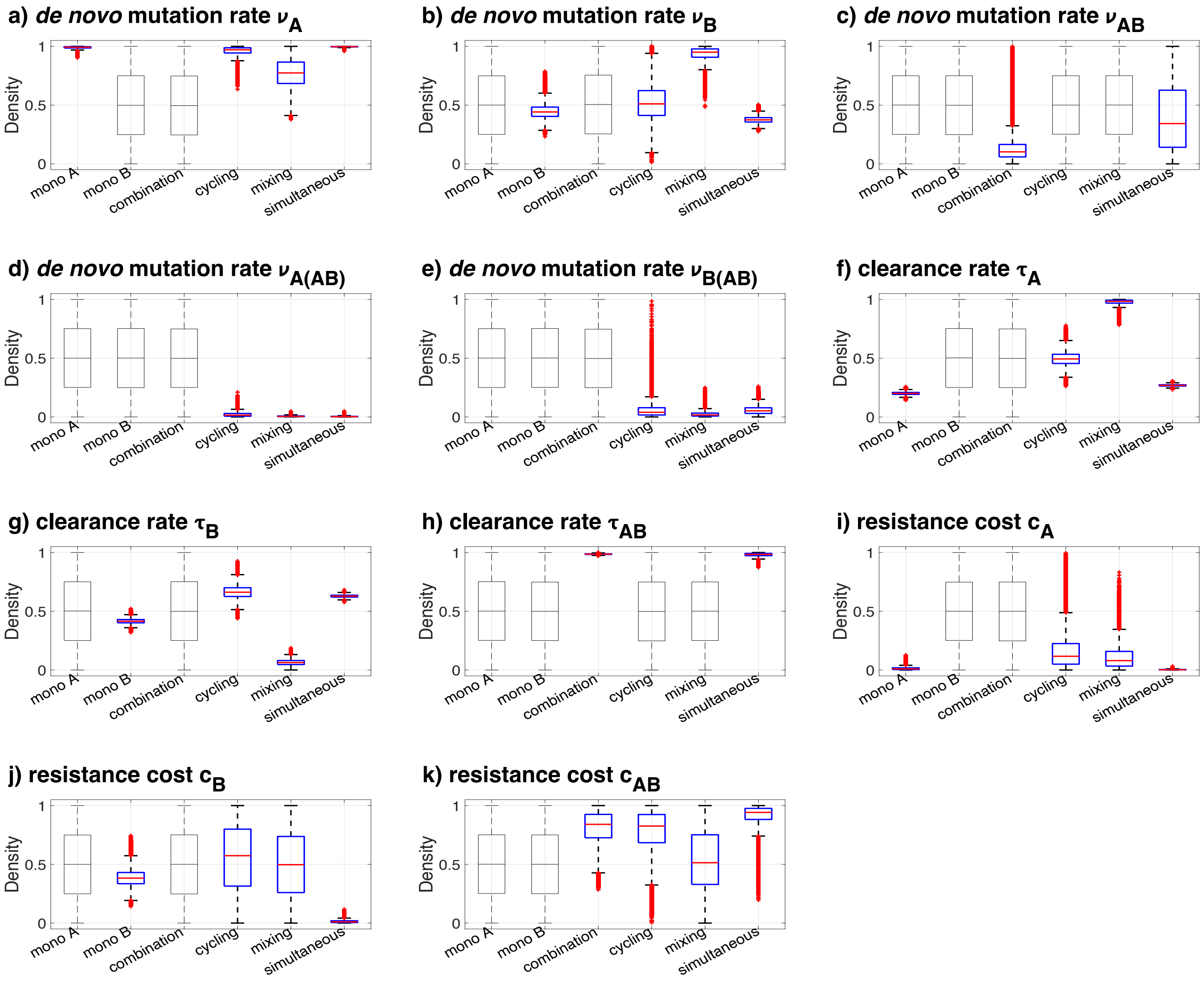

Fig. S5. Posterior distributions (estimated probability distribution for a given parameter via model fitting) obtained from the independent and simultaneous estimation. (a)-(k) Posterior distributions of each parameter for independent estimation using the data for different treatment strategies and simultaneous estimation. Only the relevant parameters for a given treatment strategy are coloured. Parameters irrelevant to the corresponding treatment strategy and thus cannot be identified are shown as thin box plots, and equal to their prior distribution, which is uniform between 0 and 1.

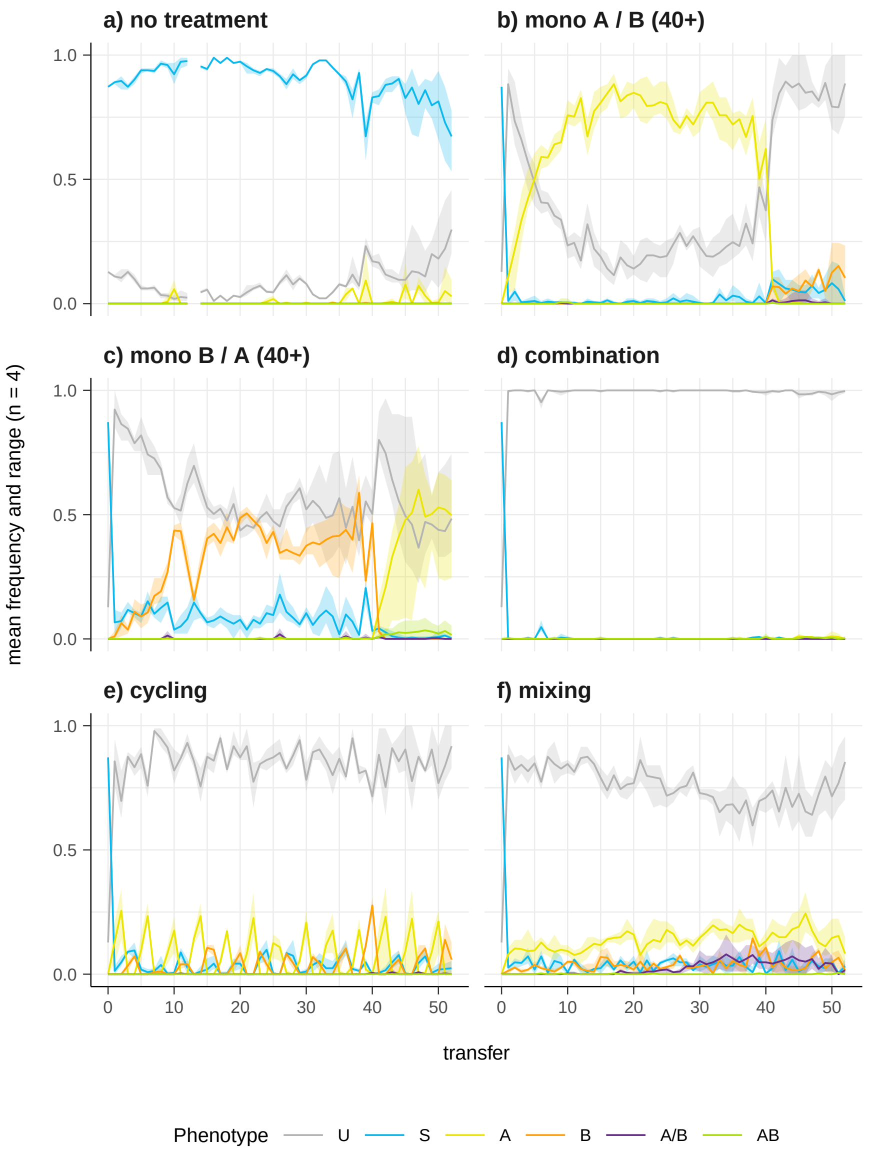

Fig. S6. Phenotype frequencies during experimental evolution in the absence of preexisting resistance (Scenario ∅). Ribbons indicate observed range in 4 replicate populations and lines denote the mean. (a) No treatment. Gap at transfer 13 is due to missing data resulting from a mechanical malfunction of the setup. (b) Monotherapy A (nalidixic acid), after transfer 40 switch to monotherapy B (streptomycin). (c) Monotherapy B (streptomycin), after transfer 40 switch to monotherapy A (nalidixic acid). (d) Combination therapy. (e) Cycling, treatment is switched every 2 transfers between nalidixic acid and streptomycin. (f) Mixing, treatment with nalidixic acid or streptomycin is randomly assigned to each well for each transfer. Each antibiotic is used in all treatments at 2 × MIC.

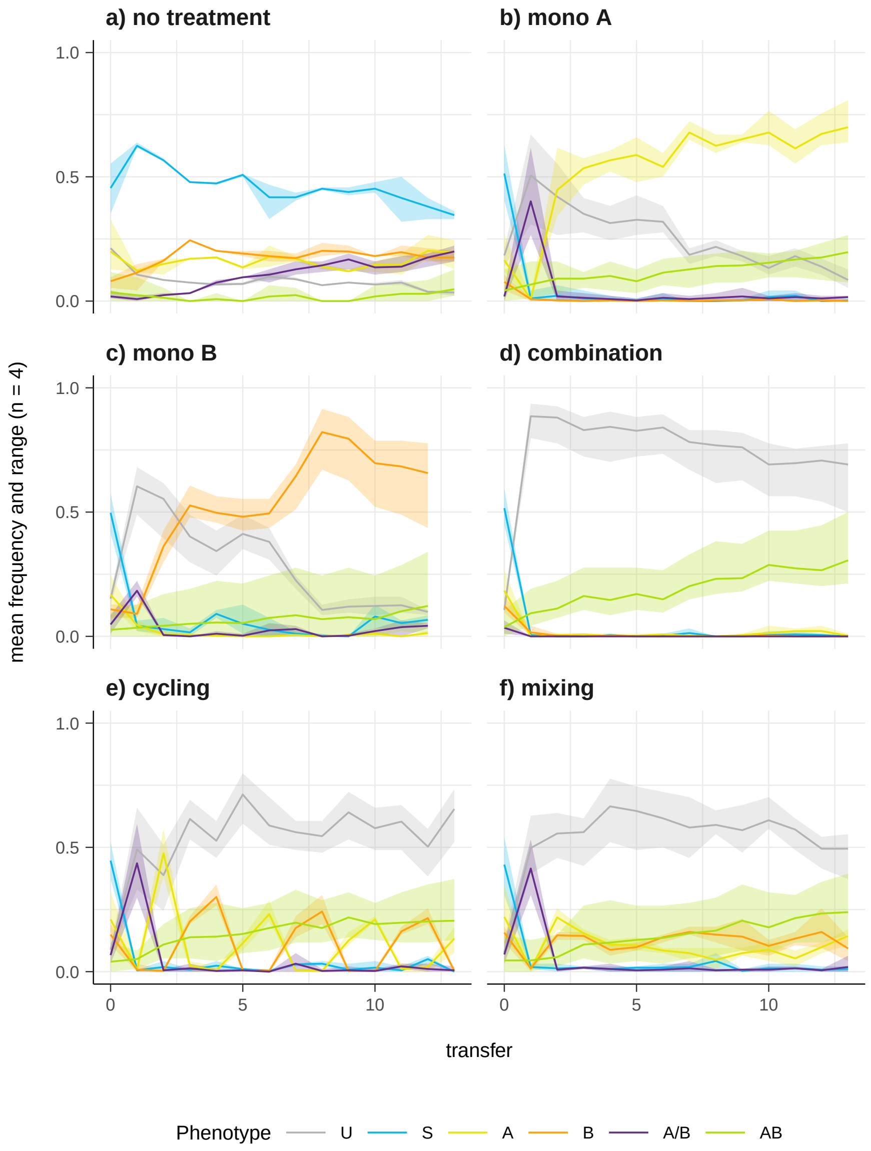

Fig. S7. Phenotype frequencies during experimental evolution with inflow of both single resistances (Scenario 1). Ribbons indicate observed range in 4 replicate populations and lines denote the mean. (a) No treatment. (b) Monotherapy A (nalidixic acid). (c) Monotherapy B (streptomycin). (d) Combination therapy. (e) Cycling, treatment is switched every 2 transfers between nalidixic acid streptomycin. (f) Mixing, treatment with nalidixic acid or streptomycin is randomly assigned to each well for each transfer. Each antibiotic is used in all treatments at 2 × MIC.

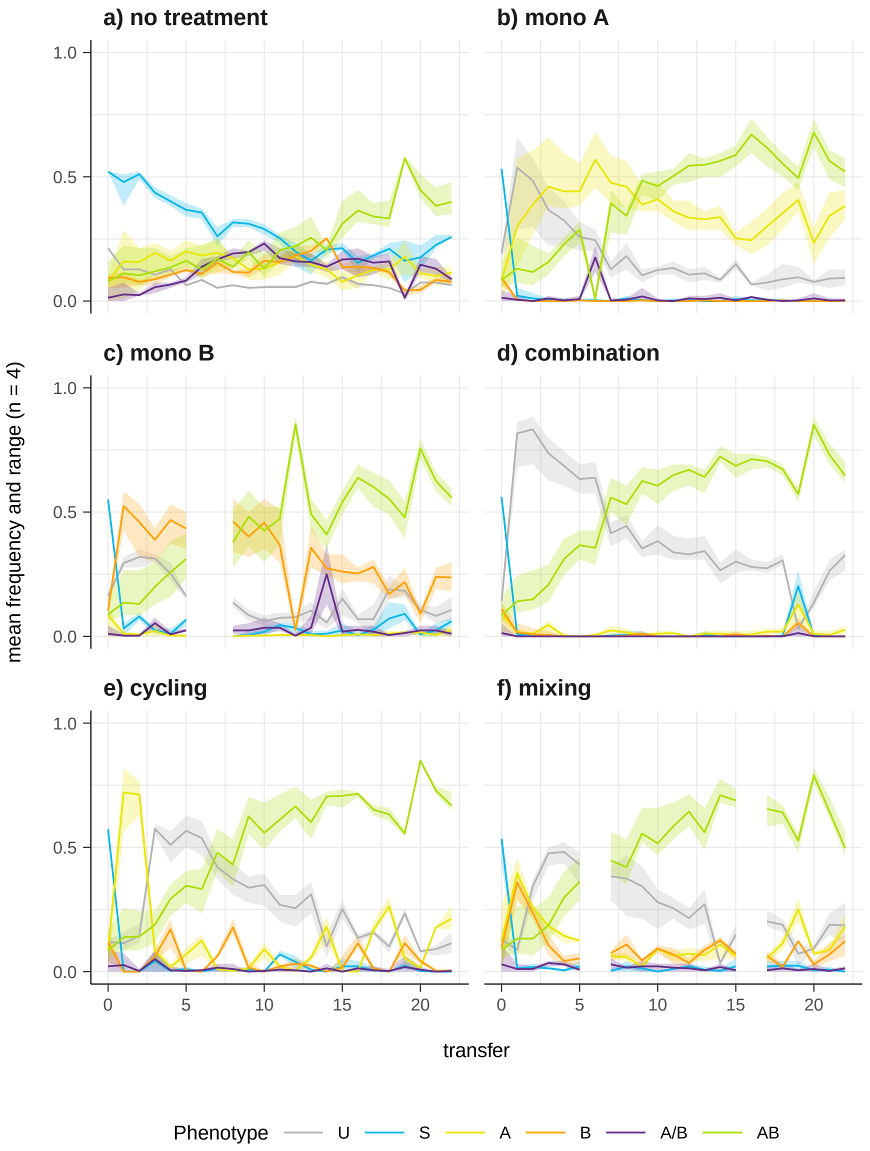

Fig. S8. Phenotype frequencies during experimental evolution with inflow of both single and double resistance (Scenario 2). Ribbons indicate observed range in 4 replicate populations and lines denote the mean. Gaps are due to missing data resulting from a mechanical malfunction of the setup. (a) No treatment. (b) Monotherapy A (nalidixic acid). (c) Monotherapy B (streptomycin). (d) Combination therapy. (e) Cycling, treatment is switched every 2 transfers between nalidixic acid streptomycin. (f) Mixing, treatment with nalidixic acid or streptomycin is randomly assigned to each well for each transfer. Each antibiotic is used in all treatments at 2 $\times$ MIC.

a)

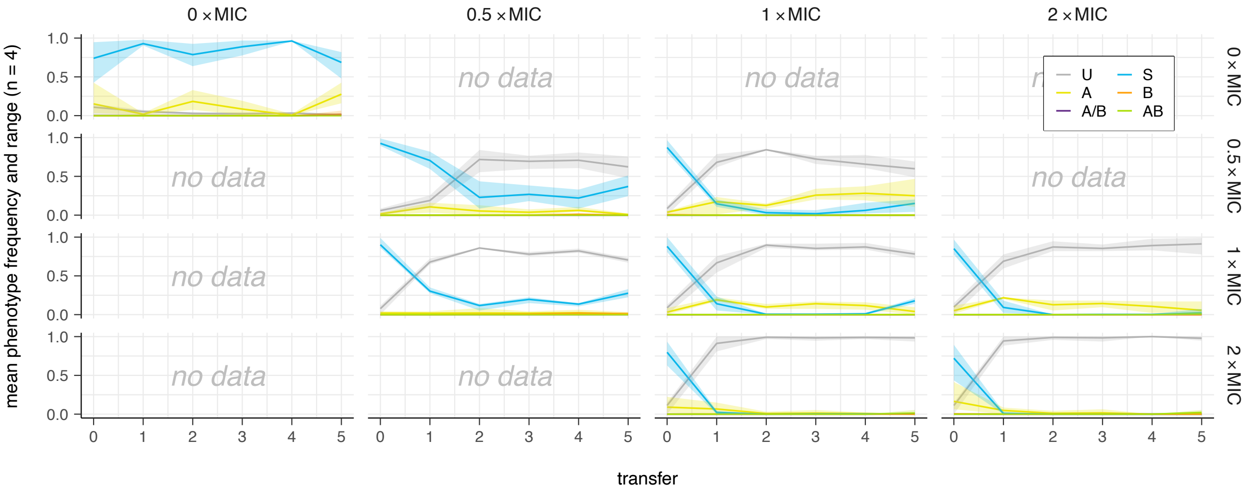

b)

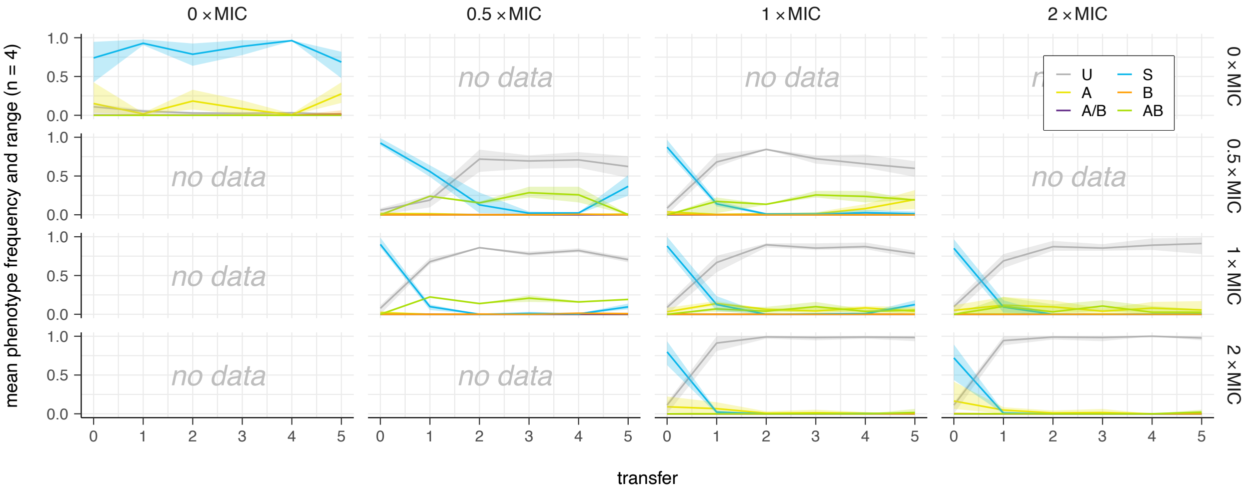

Fig. S9. Phenotype frequencies during experimental evolution in the absence of preexisting resistance at different concentrations of streptomycin and nalidixic acid. Ribbons indicate observed range in 4 replicate populations and lines denote the mean. Concentrations are indicated as multiples of MIC (MIC for streptomycin: 6.25µg/ml; MIC for nalidixic acid: 10µg/ml). (a) Frequency of resistance to high concentration of drugs used on plate (10 × MIC) while panel (b) shows resistance to the drug concentration used in liquid culture.

Table S2. Mutations in resistance determinant regions (RDR) of randomly picked populations in Scenario ∅. Brackets denote ambiguous sequencing results indicative of a mixed population.

Strategy	RDR	Rep.	timepoint	ID	Mutation	AA change
mono A	gyrA	1	30	2-30-C11-gyrA	A260G	D87G
mono A	gyrA	1	30	2-30-G3-gyrA	A260G	D87G
mono A	gyrA	1	30	2-30-K1-gyrA	G259A	D87N
mono A	gyrA	2	30	2-30-G10-gyrA	A260G	D87G
mono A	gyrA	2	30	2-30-G24-gyrA	A260G	D87G
mono A	gyrA	2	30	2-30-M12-gyrA	(T247C)	(S83P)
mono A	gyrA	3	30	2-30-D2-gyrA	A260G	D87G
mono A	gyrA	3	30	2-30-F18-gyrA	A260G	D87G
mono A	gyrA	3	30	2-30-N16-gyrA	A260G	D87G
mono A	gyrA	4	30	2-30-D9-gyrA	G259A	D87N
mono A	gyrA	4	30	2-30-F23-gyrA	A260G	D87G
mono A	gyrA	4	30	2-30-H13-gyrA	(G259A), (A260G)	(D87G, D87N or D87S)
mono B	rpsL	1	30	3-30-C17-rpsL	*no mutation*	
mono B	rpsL	1	30	3-30-E7-rpsL	A263G	K88G
mono B	rpsL	1	30	3-30-O9-rpsL	A128G	K43R
mono B	rpsL	2	30	3-30-C2-rpsL	A263G	K88G
mono B	rpsL	2	30	3-30-E12-rpsL	A263G	K88G
mono B	rpsL	2	30	3-30-O10-rpsL	A263G	K88G
mono B	rpsL	3	30	3-30-D2-rpsL	A263G	K88G
mono B	rpsL	3	30	3-30-H6-rpsL	A129C	K43N
mono B	rpsL	3	30	3-30-J10-rpsL	A263G	K88G
mono B	rpsL	4	30	3-30-D11-rpsL	A262G	K88E
mono B	rpsL	4	30	3-30-F11-rpsL	A263G	K88G
mono B	rpsL	4	30	3-30-H13-rpsL	A128G	K43R
cycling	gyrA	1	30	5-30-A23-gyrA	A260G	D87G
cycling	gyrA	1	30	5-30-K7-gyrA	A260G	D87G
cycling	gyrA	1	30	5-30-O3-gyrA	A260G	D87G
cycling	gyrA	2	30	5-30-E6-gyrA	A260G	D87G
cycling	gyrA	2	30	5-30-G2-gyrA	A260G	D87G
cycling	gyrA	2	30	5-30-O10-gyrA	A260G	D87G
cycling	gyrA	3	30	5-30-F4-gyrA	A260G, (C248T)	D87G, (S83L)
cycling	gyrA	3	30	5-30-J20-gyrA	A260G	D87G
cycling	gyrA	3	30	5-30-N8-gyrA	(C248T), (A260G)	(S83L), (D87G)
cycling	gyrA	4	30	5-30-B1-gyrA	(C248T), (A260G)	(S83L), (D87G)
cycling	gyrA	4	30	5-30-B23-gyrA	A260G	D87G
cycling	gyrA	4	30	5-30-N7-gyrA	A260G	D87G
cycling	rpsL	1	20	5-20-A17-rpsL	A263G	K88G
cycling	rpsL	1	20	5-20-A21-rpsL	A263G	K88G
cycling	rpsL	1	20	5-20-C15-rpsL	A263G	K88G
cycling	rpsL	2	20	5-20-A18-rpsL	(A263G)	(K88G)
cycling	rpsL	2	20	5-20-E18-rpsL	A263G	K88G
cycling	rpsL	2	20	5-20-M22-rpsL	A263G	K88G
cycling	rpsL	3	20	5-20-B18-rpsL	A263G	K88G
cycling	rpsL	3	20	5-20-B22-rpsL	A263G	K88G
cycling	rpsL	3	20	5-20-F18-rpsL	A263G	K88G
cycling	rpsL	4	20	5-20-B17-rpsL	A263G	K88G
cycling	rpsL	4	20	5-20-B15-rpsL	A263G	K88G
cycling	rpsL	4	20	5-20-B21-rpsL	A128G	K43R
mixing	gyrA	1	30	6-30-C11-gyrA	(C248T), A260G	(S83L), D87G
mixing	gyrA	1	30	6-30-I3-gyrA	G244A	D82N
mixing	gyrA	1	30	6-30-I11-gyrA	A260G	D87G
mixing	gyrA	2	30	6-30-C14-gyrA	A260G	D87G
mixing	gyrA	2	30	6-30-M8-gyrA	A260G	D87G
mixing	gyrA	2	30	6-30-O18-gyrA	C248T	S83L
mixing	gyrA	3	30	6-30-F6-gyrA	A260G	D87G

Table S2. **Mutations in resistance determinant regions of randomly picked populations in Scenario ∅.** ***(continued)***

Strategy	RDR	Rep.	timepoint	ID	Mutation	AA change
mixing	gyrA	3	30	6-30-H12-gyrA	A260G	D87G
mixing	gyrA	3	30	6-30-N4-gyrA	A260G	D87G
mixing	gyrA	4	30	6-30-D11-gyrA	A260G	D87G
mixing	gyrA	4	30	6-30-N9-gyrA	A260G	D87G
mixing	gyrA	4	30	6-30-P19-gyrA	A260G	D87G
mixing	rpsL	1	40	6-40-E17-rpsL	A128C	K43T
mixing	rpsL	1	40	*population could not be revived*		
mixing	rpsL	1	40	*population could not be revived*		
mixing	rpsL	2	40	6-40-E18-rpsL	A128C	K43T
mixing	rpsL	2	40	6-40-I18-rpsL	A128G	K43R
mixing	rpsL	2	40	6-40-E16-rpsL	(A128C)	(K43T)
mixing	rpsL	3	40	6-40-F18-rpsL	A128C	K43T
mixing	rpsL	3	40	6-40-J18-rpsL	A128G	K43R
mixing	rpsL	3	40	6-40-J16-rpsL	A128G	K43R
mixing	rpsL	4	40	6-40-F17-rpsL	A128C	K43T
mixing	rpsL	4	40	6-40-J17-rpsL	A128G	K43R
mixing	rpsL	4	40	6-40-L1-rpsL	*no mutation*	

Table S3. Strains used in this study.

Strain	Genotype	Phenotype	Reference
MG1655	*Escherichia coli* F- lambda-, rph-1		
MDS42(YFP)	*E. coli* MDS42 *rihB*::(YFP-*tetR-cat*)	(Cm^R)	gift from Csaba Pál; Pósfai et al. (7), Fehér et al.(8)
JW2703	*E. coli* BW25113 △mutS::FRT-kan-FRT	(Kan^R)	Baba et al.(9)
S	MG1655 *rihB*::(YFP-*tetR-cat*) △mutS::FRT-kan-FRT	S (Cm^R, Kan^R)	(this study)
NalR	wt *gyrA*S83L	A (Nal^R, Cm^R, Kan^R)	(this study)
SmR	wt *rpsL*K43R	B (Sm^R, Cm^R, Kan^R)	(this study)
NalR-SmR	wt *rpsL*K43R *gyrA*S83L	AB (Nal^R, Sm^R, Cm^R, Kan^R)	(this study)

Cm^R: Chloramphenicol resistance; Kan^R: Kanamycin resistance; Nal^R: Nalidixic acid resistance; Sm^R: Streptomycin resistance.
Please refer to main text for details on phenotype definitions.

Statistical tables

Table S4. Scenario 0: Effect of treatment strategy on the frequency of uninfected and resistant populations (ANOVA).

	Df	Sum Sq	Mean Sq	F	Pr(> F)
p	2	2.427	1.213475	3065	< 0.001
plate	5	0	0	0	< 0.001
p:plate	10	7.5657	0.756567	1911	< 0.001
Residuals	54	0.0214	0.000396		< 0.001

Table S5. Scenario 0: Multiple comparison of phenotype frequencies between treatment strategies.

Phenotype	Linear Hypothesis	Estimate	Std. Error	Pr(> $\|t\|$)
U	2 − 1 == 0	0.222	0.0141	< 0.001
U	3 − 1 == 0	0.534	0.0141	< 0.001
U	4 − 1 == 0	0.973	0.0141	< 0.001
U	5 − 1 == 0	0.858	0.0141	< 0.001
U	6 − 1 == 0	0.814	0.0141	< 0.001
U	3 − 2 == 0	0.312	0.0141	< 0.001
U	4 − 2 == 0	0.751	0.0141	< 0.001
U	5 − 2 == 0	0.636	0.0141	< 0.001
U	6 − 2 == 0	0.592	0.0141	< 0.001
U	4 − 3 == 0	0.439	0.0141	< 0.001
U	5 − 3 == 0	0.324	0.0141	< 0.001
U	6 − 3 == 0	0.28	0.0141	< 0.001
U	5 − 4 == 0	-0.115	0.0141	< 0.001
U	6 − 4 == 0	-0.159	0.0141	< 0.001
U	6 − 5 == 0	-0.0439	0.0141	0.0925
S	2 − 1 == 0	-0.955	0.0141	< 0.001
S	3 − 1 == 0	-0.88	0.0141	< 0.001
S	4 − 1 == 0	-0.956	0.0141	< 0.001
S	5 − 1 == 0	-0.928	0.0141	< 0.001
S	6 − 1 == 0	-0.924	0.0141	< 0.001
S	3 − 2 == 0	0.0758	0.0141	< 0.001
S	4 − 2 == 0	-0.000665	0.0141	1
S	5 − 2 == 0	0.0279	0.0141	0.721
S	6 − 2 == 0	0.0312	0.0141	0.549
S	4 − 3 == 0	-0.0765	0.0141	< 0.001
S	5 − 3 == 0	-0.0479	0.0141	0.0441
S	6 − 3 == 0	-0.0445	0.0141	0.0825
S	5 − 4 == 0	0.0286	0.0141	0.688
S	6 − 4 == 0	0.0319	0.0141	0.515
S	6 − 5 == 0	0.00332	0.0141	1
R	2 − 1 == 0	0.733	0.0141	< 0.001
R	3 − 1 == 0	0.346	0.0141	< 0.001
R	4 − 1 == 0	-0.0166	0.0141	0.995
R	5 − 1 == 0	0.0698	0.0141	< 0.001
R	6 − 1 == 0	0.11	0.0141	< 0.001
R	3 − 2 == 0	-0.388	0.0141	< 0.001
R	4 − 2 == 0	-0.75	0.0141	< 0.001
R	5 − 2 == 0	-0.664	0.0141	< 0.001
R	6 − 2 == 0	-0.623	0.0141	< 0.001
R	4 − 3 == 0	-0.362	0.0141	< 0.001

Table S5. Scenario 0: Multiple comparison of phenotype frequencies between treatment strategies. ***(continued)***

Phenotype	Linear Hypothesis	Estimate	Std. Error	Pr(> |t|)
R	5 − 3 == 0	-0.276	0.0141	< 0.001
R	6 − 3 == 0	-0.235	0.0141	< 0.001
R	5 − 4 == 0	0.0864	0.0141	< 0.001
R	6 − 4 == 0	0.127	0.0141	< 0.001
R	6 − 5 == 0	0.0406	0.0141	0.163

Table S6. Scenario I: Effect of treatment strategy on the frequency of uninfected and resistant populations (ANOVA).

	Df	Sum Sq	Mean Sq	F	Pr(> F)
p	2	2.605	1.30253	582	< 0.001
plate	5	0	0	0	< 0.001
p:plate	10	3.24	0.32398	145	< 0.001
Residuals	54	0.121	0.00224		< 0.001

Table S7. Scenario I: Multiple comparison of phenotype frequencies between treatment strategies.

Phenotype	Linear Hypothesis	Estimate	Std. Error	Pr(> |t|)
U	2 − 1 == 0	0.0951	0.0335	0.178
U	3 − 1 == 0	0.0532	0.0335	0.928
U	4 − 1 == 0	0.651	0.0335	< 0.001
U	5 − 1 == 0	0.518	0.0335	< 0.001
U	6 − 1 == 0	0.498	0.0335	< 0.001
U	3 − 2 == 0	-0.0419	0.0335	0.991
U	4 − 2 == 0	0.556	0.0335	< 0.001
U	5 − 2 == 0	0.423	0.0335	< 0.001
U	6 − 2 == 0	0.403	0.0335	< 0.001
U	4 − 3 == 0	0.598	0.0335	< 0.001
U	5 − 3 == 0	0.465	0.0335	< 0.001
U	6 − 3 == 0	0.445	0.0335	< 0.001
U	5 − 4 == 0	-0.133	0.0335	0.0082
U	6 − 4 == 0	-0.153	0.0335	0.00119
U	6 − 5 == 0	-0.0199	0.0335	1
S	2 − 1 == 0	-0.412	0.0335	< 0.001
S	3 − 1 == 0	-0.372	0.0335	< 0.001
S	4 − 1 == 0	-0.417	0.0335	< 0.001
S	5 − 1 == 0	-0.402	0.0335	< 0.001
S	6 − 1 == 0	-0.412	0.0335	< 0.001
S	3 − 2 == 0	0.0399	0.0335	0.994
S	4 − 2 == 0	-0.00532	0.0335	1
S	5 − 2 == 0	0.00997	0.0335	1
S	6 − 2 == 0	-0.000665	0.0335	1
S	4 − 3 == 0	-0.0452	0.0335	0.981
S	5 − 3 == 0	-0.0299	0.0335	1
S	6 − 3 == 0	-0.0406	0.0335	0.993
S	5 − 4 == 0	0.0153	0.0335	1
S	6 − 4 == 0	0.00465	0.0335	1
S	6 − 5 == 0	-0.0106	0.0335	1

Table S7. Scenario I: Multiple comparison of phenotype frequencies between treatment strategies. ***(continued)***

Phenotype	Linear Hypothesis	Estimate	Std. Error	$\Pr(>\|t\|)$
R	$2 - 1 == 0$	0.316	0.0335	< 0.001
R	$3 - 1 == 0$	0.318	0.0335	< 0.001
R	$4 - 1 == 0$	-0.234	0.0335	< 0.001
R	$5 - 1 == 0$	-0.116	0.0335	0.0355
R	$6 - 1 == 0$	-0.0858	0.0335	0.316
R	$3 - 2 == 0$	0.00199	0.0335	1
R	$4 - 2 == 0$	-0.551	0.0335	< 0.001
R	$5 - 2 == 0$	-0.433	0.0335	< 0.001
R	$6 - 2 == 0$	-0.402	0.0335	< 0.001
R	$4 - 3 == 0$	-0.553	0.0335	< 0.001
R	$5 - 3 == 0$	-0.435	0.0335	< 0.001
R	$6 - 3 == 0$	-0.404	0.0335	< 0.001
R	$5 - 4 == 0$	0.118	0.0335	0.0318
R	$6 - 4 == 0$	0.148	0.0335	0.00182
R	$6 - 5 == 0$	0.0306	0.0335	1

Table S8. Scenario II: Effect of treatment strategy on the frequency of uninfected and resistant populations (ANOVA).

	Df	Sum Sq	Mean Sq	F	$\Pr(> F)$
p	2	6.4522	3.226119	3478.9	< 0.001
plate	5	0	0	0	< 0.001
p:plate	10	0.7891	0.078912	85.1	< 0.001
Residuals	54	0.0501	0.000927		< 0.001

Table S9. Scenario II: Multiple comparison of phenotype frequencies between treatment strategies.

Phenotype	Linear Hypothesis	Estimate	Std. Error	$\Pr(>\|t\|)$
U	$2 - 1 == 0$	0.0618	0.0215	0.167
U	$3 - 1 == 0$	0.0193	0.0215	1
U	$4 - 1 == 0$	0.296	0.0215	< 0.001
U	$5 - 1 == 0$	0.247	0.0215	< 0.001
U	$6 - 1 == 0$	0.218	0.0215	< 0.001
U	$3 - 2 == 0$	-0.0426	0.0215	0.727
U	$4 - 2 == 0$	0.234	0.0215	< 0.001
U	$5 - 2 == 0$	0.186	0.0215	< 0.001
U	$6 - 2 == 0$	0.156	0.0215	< 0.001
U	$4 - 3 == 0$	0.277	0.0215	< 0.001
U	$5 - 3 == 0$	0.228	0.0215	< 0.001
U	$6 - 3 == 0$	0.199	0.0215	< 0.001
U	$5 - 4 == 0$	-0.0485	0.0215	0.525
U	$6 - 4 == 0$	-0.0778	0.0215	0.0241
U	$6 - 5 == 0$	-0.0293	0.0215	0.98
S	$2 - 1 == 0$	-0.261	0.0215	< 0.001
S	$3 - 1 == 0$	-0.237	0.0215	< 0.001
S	$4 - 1 == 0$	-0.262	0.0215	< 0.001
S	$5 - 1 == 0$	-0.234	0.0215	< 0.001
S	$6 - 1 == 0$	-0.252	0.0215	< 0.001

Table S9. Scenario II: Multiple comparison of phenotype frequencies between treatment strategies. ***(continued)***

Phenotype	Linear Hypothesis	Estimate	Std. Error	Pr(> $\|t\|$)
S	$3 - 2 == 0$	0.0239	0.0215	0.997
S	$4 - 2 == 0$	-0.000665	0.0215	1
S	$5 - 2 == 0$	0.0273	0.0215	0.99
S	$6 - 2 == 0$	0.00931	0.0215	1
S	$4 - 3 == 0$	-0.0246	0.0215	0.996
S	$5 - 3 == 0$	0.00332	0.0215	1
S	$6 - 3 == 0$	-0.0146	0.0215	1
S	$5 - 4 == 0$	0.0279	0.0215	0.987
S	$6 - 4 == 0$	0.00997	0.0215	1
S	$6 - 5 == 0$	-0.018	0.0215	1
R	$2 - 1 == 0$	0.199	0.0215	< 0.001
R	$3 - 1 == 0$	0.218	0.0215	< 0.001
R	$4 - 1 == 0$	-0.0339	0.0215	0.933
R	$5 - 1 == 0$	-0.0133	0.0215	1
R	$6 - 1 == 0$	0.0339	0.0215	0.933
R	$3 - 2 == 0$	0.0186	0.0215	1
R	$4 - 2 == 0$	-0.233	0.0215	< 0.001
R	$5 - 2 == 0$	-0.213	0.0215	< 0.001
R	$6 - 2 == 0$	-0.166	0.0215	< 0.001
R	$4 - 3 == 0$	-0.252	0.0215	< 0.001
R	$5 - 3 == 0$	-0.231	0.0215	< 0.001
R	$6 - 3 == 0$	-0.184	0.0215	< 0.001
R	$5 - 4 == 0$	0.0206	0.0215	0.999
R	$6 - 4 == 0$	0.0678	0.0215	0.0859
R	$6 - 5 == 0$	0.0472	0.0215	0.571

References

1. DW Marquardt, An algorithm for least-squares estimation of nonlinear parameters. *J Soc Indust Appl Math* **11**, 431–441 (1963).
2. S Chib, E Greenberg, Understanding the metropolis-hastings algorithm. *Am Stat* **49**, 327–335 (1995).
3. B Tepekule, H Uecker, I Derungs, A Frenoy, S Bonhoeffer, Modeling antibiotic treatment in hospitals: A systematic approach shows benefits of combination therapy over cycling, mixing, and mono-drug therapies. *PLoS Comp Biol* **13**, e1005745 (2017).
4. SE Luria, M Delbrück, Mutations of Bacteria from Virus Sensitivity to Virus Resistance. *Genetics* **28**, 491–511 (1943).
5. MA Kohanski, MA Depristo, JJ Collins, Sublethal Antibiotic Treatment Leads to Multidrug Resistance via Radical-Induced Mutagenesis. *Mol. Cell* **37**, 311–320 (2010).
6. A Mazoyer, R Drouilhet, S Despreaux, B Ycart, flan: An R Package for Inference on Mutation Models. *The R Journal* **9**, 334–351 (2017).
7. G Pósfai, et al., Emergent Properties of Reduced-Genome Escherichia coli. *Science* **312**, 1044–1046 (2006).
8. T Feher, et al., Competition between Transposable Elements and Mutator Genes in Bacteria. *Mol. Biol. Evol.* **29**, 3153–3159 (2012).
9. T Baba, et al., Construction of Escherichia coli K-12 in-frame, single-gene knockout mutants: the Keio collection. *Mol Syst Biol* **2**, 2006.0008 (2006).
